# Nasal Delivery Devices: A Comparative Study on Cadaver Model

**DOI:** 10.1155/2019/4602651

**Published:** 2019-03-28

**Authors:** Antonio Moffa, Andrea Costantino, Vittorio Rinaldi, Lorenzo Sabatino, Eleonora Maria Consiglia Trecca, Peter Baptista, Paolo Campisi, Michele Cassano, Manuele Casale

**Affiliations:** ^1^Unit of Otolaryngology, University of Foggia, Foggia, Italy; ^2^Unit of Otolaryngology, UOS ORL TI, Campus Bio-Medico University, Rome, Italy; ^3^Unit of Otolaryngology, Clinica Universitaria de Navarra, Pamplona, Spain; ^4^Department of Otolaryngology-Head and Neck Surgery, University of Toronto, 190 Elizabeth Street 3S-438, Toronto M5G 2C4, Canada

## Abstract

Nasal nebulization is a more effective method of delivering topical medication than nasal spray. The purpose of this study was to assess the deposition patterns of nebulization in delivering topical agents to the nasal cavities in the human cadaveric model using a color-based method. We have compared these following nasal devices: single-dose vial irrigation, syringe-irrigation, common nasal spray, Spray-sol, MAD nasal, and Rinowash nasal douche. Endoscopic images were recorded at six anatomical regions prior to and following each nasal device application and four reviewers evaluated the amount of surface area staining. At the nasal vestibule, the blue dye distribution achieved with Spray-sol was more extensive than nasal sprays. At inferior turbinate and nasal cavity floor, single dose vial, syringe, MAD nasal, Spray-sol, and Rinowash demonstrated a greater extent of dye distribution than nasal spray. At the middle turbinate, the average score of both Spray-sol and MAD nasal was significantly higher than other nasal investigated devices. At the nasopharynx, Spray-sol nebulization covers a surface significantly greater than other devices. Compared to traditional sprays, Spray-sol and MAD nasal provided a more effective method of delivering topical agents to the deeper and higher portions of the nasal cavities.

## 1. Introduction

Medical management of sinonasal diseases increasingly involves the use of topical agents, which offer an improved ability to deliver high concentrations of drugs to the nasal mucosa avoiding systemic effects [1]. Given the pivotal role that topical therapies play in many sinonasal conditions such as acute rhinosinusitis (ARS) and chronic rhinosinusitis (CRS), allergic and nonallergic rhinitis, considerable interest in the effectiveness, tolerability, and compliance of specific nasal devices has become evident [2, 3]. Intranasal medications or other substances are usually administered by nasal drops and syringes, common nasal spray, and nebulizers [4]. The success of topical nasal treatment depends on multiple factors such as delivery methods, gravity, obstructing anatomical structures, head positions, and viscosity of administrated substance. Nebulized particle size plays an important role in airway deposition: particle diameter of 1-5 *μ*m is good for the lower airway; from 5 to 10 *μ*m particles deposit mostly in the trachea and bronchi, while diameter > 10 *μ*m particles deposit mostly in the nose [5, 6]. A recent comparative study [7] showed that Spray-sol produced particles with average diameter (D) of 16 *μ*m similar to the aeroassisted nebulizer (Rinowash type, D=16.5 *μ*m), but significantly smaller than those produced by nasal spray with pressurized canister (D=59.3 *μ*m) and nasal spray with a pump bottle (D= 34.7 *μ*m).

Many studies suggested that nasal nebulization is a more effective method of delivering topical medication than nasal spray because it generates small, slow moving particles that traverse the nasal cavity covering a greater surface of nasal mucosa [8]. Intranasal deposition pattern of different nasal devices can be investigated with anatomical models such as cadaver heads, nasal cavity replicas or nasal casts, and other trials used in vivo by gamma camera imaging [9, 10].

The purpose of this study was to assess the deposition patterns of nebulization in delivering topical agents to the nasal cavities in the human cadaveric model. It was hypothesized that nebulization provides a more extensive and intensive delivery of solutions to the nasal cavities than sprays.

## 2. Material and Methods

This study was conducted at the ICLO Teaching and Research Center of Arezzo (Italy) in July 2018.

### 2.1. Cadaver Preparation

Cadaveric head was initially evaluated by nasal endoscopy using 0-degree and 30-degree, 3.0-mm rigid endoscopes (Karl Storz, Tuttlingen, Germany) to rule out any anatomical anomalies such as nasal septum perforation, deviated nasal septum or septal crest, aberrant nasal turbinates, and sinonasal disease such as nasal polyps or other anomalies that could interfere with the realization of this study. The head was placed in the upright position slightly inclined forward, in line with the correct position to be used by patients.

### 2.2. Administration of Treatment Solutions

Topical solution was prepared using a blue vegetable dye purchased from local grocery store. It was added in a quantity adequate to tint the solution dark blue to be visualized endoscopically but not concentrated enough to stain the sinonasal mucosa. Several trials were performed coloring the oral cavity with dye's increasing dilution in order to obtain the optimal concentration. In the definitive trial, the blue dye was diluted in a balanced saline solution at a concentration of 12.5% (1:8 dilution) [10–12]. We evaluated the following nasal devices: single-dose vial irrigation, syringe-irrigation (without needle), common nasal spray, Spray-sol, MAD nasal, and Rinowash nasal douche.

These different nasal devices are described as follows:

- Single-dose vial irrigation: each unit dose vial contains 5 ml of saline solution and has an inverted milliliter graduation on the tube for accurate dispensing.
- Syringe-irrigation: it is a common 5 ml nasal syringe without needle. Each patient aspirates the solution with the syringe and then delivers the contents into the nasal cavity.
- Nasal spray: it is a common nasal spray fitted with a metering atomising spray pump.
- Spray-sol: it is a nasal nebulizer characterized by the ability to nebulize high viscosity substances similar to an aerosol, the very low administration times (10 seconds to 5 cc of substance). It is portable and does not need electricity as it is attached to a Luer-Lok syringe [13].
- Mucosal atomization device (MAD) Nasal: it consists of an atomization nasal nozzle tip attached to standard 3 cc syringe. The patient unscrews the tip and then pulls the drug solution into the syringe by lifting the plunger; then he or she screws the tip back into place and after placing the tip in the nostril he or she depresses the plunger to dispense the drug [14].
- Rinowash nasal douche: it is a micronized nasal douche specifically designed to administer correct endonasal therapy and proves particularly effective in the treatment of the upper respiratory ways. This nasal douche is connected to the compressor device for aerosol therapy allowing a complete treatment of the upper airway in 2-3 minutes. [15, 16]. It requires the use of electricity. Each device has been used as recommended by the medication package insert. In particular, each tip was inserted at a 45-degree angle into the nasal aperture and then directed to the ipsilateral orbit. For each of these nasal devices we provided 2,5 ml of vegetable diluted dye.

We examined all six devices randomizing the administration to the cadaver specimen's nasal cavities.

### 2.3. Endoscopic Data Collection

Endoscopic videos and images were recorded at six standardized anatomical regions prior to and following each nasal device application to document staining. The anatomical regions investigated were as follows: nasal vestibule, inferior turbinate, middle turbinate, nasopharynx, nasal cavity floor, and superior olfactory cleft [17].

### 2.4. Scoring of the Extent of Distribution

In order to facilitate data analysis, images of the anatomic regions of nasal cavities before and after blue dye treatment were collated together for review. Four reviewers scored these images, using an ordinal grading scale to rate the amount of surface area staining (Table 1) [17].

### 2.5. Statistical Analysis

The results were collected and stored in a Microsoft Excel spreadsheet. Statistical analyses were performed using the statistical package STATA version 13 (StataCorp LP, College Station, TX, USA). Graphs were prepared using* GraphPad Prism *6.0 for MacBook Air (GraphPad, La Jolla, CA). The results are expressed as mean and standard deviation (SD). Starting from the average scores obtained from the four observations, we performed a two-way analysis of variance (ANOVA) considering devices and anatomical regions as independent variables. We compared the staining degree of the different anatomical regions obtained by different devices performing Tukey's test for multiple comparisons.

## 3. Results

**Figure 1:**
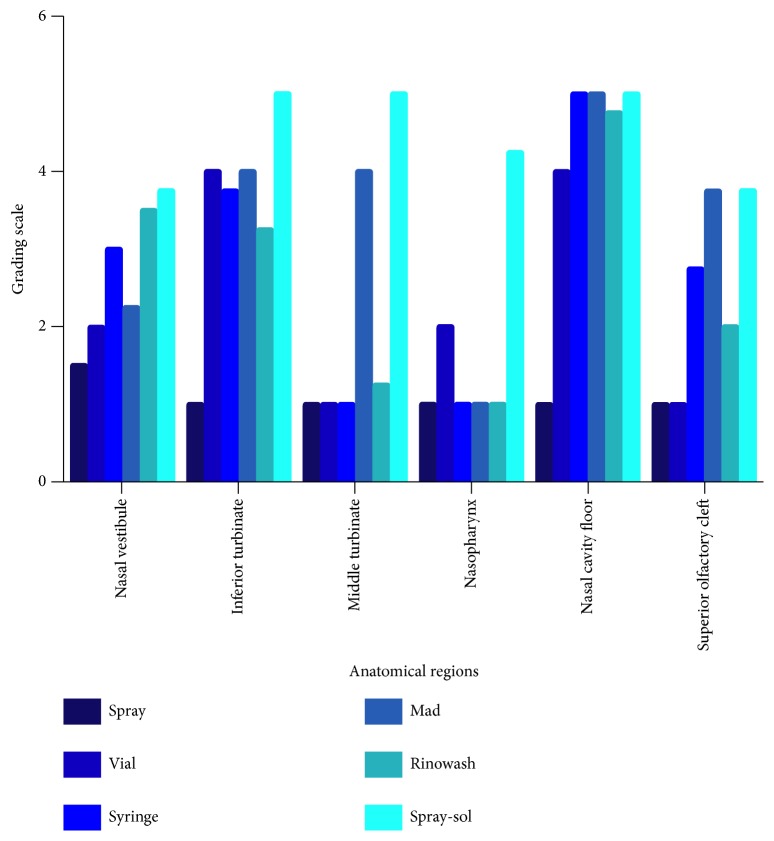
Staining average score of each device investigated for each anatomical region during the first stage of the study.

Mean scores obtained from the four reviewers during the study and related to the six anatomical regions are recorded in Figure 1. All multiple comparisons performed to compare the six devices are illustrated in Table 2.

Based on average reviewer ratings, at the nasal vestibule subsite, the blue dye distribution achieved with Spray-sol was more extensive than nasal sprays (Spray-sol 3.75±0.96; nasal spray 1.5±0.58; P<0.01).

Considering inferior turbinate (nasal spray 1±0; single dose vial 4±0; syringe 3.75±0.5; MAD nasal 4±0.82; Spray-sol 5±0 and Rinowash 3.25±0.5) and nasal cavity floor (nasal spray 1±0; single dose vial 4± 0; syringe 5±0; MAD nasal 5±0; Spray-sol 5±0 and Rinowash 4.75±0.5) subsites, the following nasal devices: single dose vial, syringe, MAD nasal, Spray-sol, and Rinowash demonstrated a greater extent of distribution than nasal spray with statistical significance (P<0.01).

**Figure 2:**
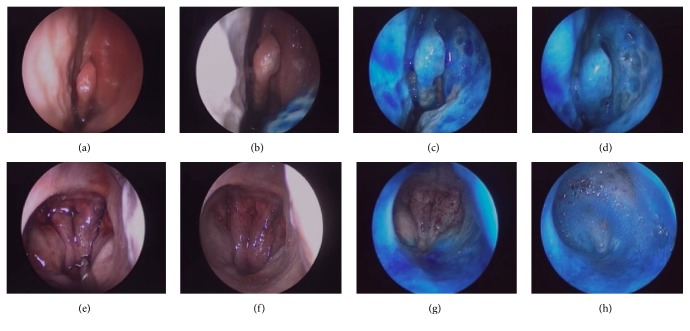
Representative endoscopic images of the left nostril during various stages of blue dye treatment. At the middle turbinate: nasal mucosa prior to treatment (a) and following nasal spray (b), MAD nasal (c), and Spray-sol (d) applications; at the nasopharynx: nasal mucosa prior to treatment (e) and following nasal spray (f), MAD nasal (g), and Spray-sol (h) applications.

At the middle turbinate, the average score of both Spray-sol (5±0) and MAD nasal (4±0) was significantly higher than other nasal investigated devices (nasal spray 1±0; single dose vial 1±0; syringe 1±0; Rinowash 1.25±0.5; P<0.01). Comparing Spray-sol and Mad nasal we noticed that the blue dye distribution pattern at this site was similar (P=0.03, Table 2, Figure 2).

At the nasopharynx, Spray-sol nebulization covers a surface significantly greater than other devices (nasal spray 1±0; single dose vial 2±0; syringe 1±0; MAD nasal 1±0; Spray-sol 4.25±0.5 and Rinowash 1±0; P<0.01, Figure 2). At the olfactory mucosa region, Spray-sol and MAD nasal showed a greater extent of blue dye distribution than nasal spray and single dose vial (nasal spray 1±0; single dose vial 1±0; syringe 2.75±1.26; MAD nasal 3.75±0.5; Spray-sol 3.75±0.5 and Rinowash 2±0; P<0.01).

## 4. Discussion

Topical drug administration into the nasal cavity has become a widely prescribed form of delivering for different topical formulation such as isotonic or hypertonic solution, hyaluronic acid, steroids, or decongestants. There are different nasal devices commercially available such as syringes, nasal sprays, and nebulizers with specific properties according to particle size and administration technique. The investigations into determining distribution pattern of different nasal devices are not simple and have been limited mostly by labor-intensive methodologies. Indeed, in scientific literature there are different types of studies from blue-dyed irrigation applied to live patients and cadavers to other used irrigations with iodinated contrast (followed by computed tomography), Technetium 99m sulfur colloid, and fluorescein [17, 18].

Our results confirm data from literature showing that nasal nebulizer provides a more extensive and intensive delivery of solution in the nasal cavities especially the deeper and higher portions of nasal cavity than nasal spray (Table 2).

In particular, two nasal nebulizers, Spray-sol and MAD nasal, seem to guarantee a homogeneous and intense coloration at the middle turbinate and ostiomeatal complex (OMC), crucial region for the drainage and ventilation of the paranasal sinuses and main target of medical and surgical CRS therapy, with a slight superiority of Spray-sol compared to MAD in the second stage of the study (P<0.01, Figure 2) [19].

In addition, Spray-Sol reaches more easily the nasopharynx subsite than the other devices investigated with a statistical significance (Table 2, Figure 2) and it could be considered a useful device for adenoiditis and Eustachian tube dysfunction therapies.

The greater distributive efficacy of the nasal nebulizers may be explained by the administration of a larger solution with high positive pressure and by the smaller particles diameter than spray (2.5ml to each nostril versus 2 sprays (0.1ml x 2) at each dosing). Indeed nasal sprays typically generate particles between 50 and 100 *μ*m in diameter, and amounts between 70 and 150 *μ*L are administered per puff [20]. Conversely, Spray-sol and MAD nasal can deliver larger amount of solution for each application (5 ml and 3 ml, resp.) and produce particles with smaller diameter (16 *μ*m and 30-100 *μ*m).

Despite the better deposition pattern of the nebulizers, nasal spray is the dominant delivery device in the nasal drug delivery market, being inexpensive and simple to use. It represents the first line of treatment in many nasal conditions, which typically reaches the nasal valve area, the region of maximum resistance to airflow [21].

We have also investigated syringes and single dose vials, commonly employed to perform nasal irrigations. The positive pressure and high volume have the function of washing the floor of the nasal fossa clearing pollutants, inflammatory products, mucus, antigen, and bacteria/biofilms. However, these nasal devices may not be appropriate for drug delivery since complete sinus delivery, prolonged mucosal contact time with local absorption, and minimal depletion are often the desired properties [22].

Despite the revealing findings in this original study, clinical generalizations are limited by the use of cadaveric specimens. The cadaveric model rules out the “force” of breathing and mucociliary clearance on the distribution pattern of particles in the nasal cavities, independently from the utilized device.

Cadaveric studies also neglect important patient-related variables, which in the case of topical agent administration include patient comfort and proper usage of delivery devices.

An additional limitation inherent in this study's methodology resulted from the irreversible manner of methylene blue staining. Following administration of sprays, residual staining could not be entirely removed from the specimens with rinsing of water. Such a limitation is expected to overestimate the extent and intensity of the nebulization method as compared to the spray technique, but as noted in the included endoscopic images the differences between devices are still undeniable.

We used only one cadaver head as a consequence of limited availability and costs; the intersubject variability related with nasal cavity anatomy represents undoubtedly a factor able to modulate medication distribution; although the use of fluorescein is well validated for determining distribution within the sinonasal cavity, we used a routinely semiquantitative means to characterize agent staining intensity, the cadaveric study.

## 5. Conclusion

This study evaluated the effect of nebulization on the nasal distribution in comparison to nasal spray. According to the recent literature, nasal nebulizers showed some advantages over the traditional nasal spray in the distribution of fluorescein-impregnated saline solution, especially for the deeper and higher portions of the nasal cavities, even if nasal spray remains the most commonly used device by patients for its easy and rapid administration and many different formulations are available in this format [23]. Nebulization method could be particularly useful for ARS and CRS treatment, such as sinonasal olfactory dysfunction, adenoid hypertrophy, and Eustachian tube dysfunction. The physician should customize the topical medication delivery method on patient disease and severity of symptoms, not forgetting to explain to the patient the correct position to use during the delivery.

## Figures and Tables

**Table 1:** Grading scale.

Score	Amount of surface area staining
1	0% to 20% of subsite surface area with staining
2	21% to 40% of subsite surface area with staining
3	41% to 60% of subsite surface area with staining
4	61% to 80% of subsite surface area with staining
5	81% to 100% of subsite surface area with staining

**(a):** 

	NASAL VESTIBULE			INFERIOR TURBINATE			MIDDLE TURBINATE		
	Mean 1	Mean 2	Adjusted P value	Mean 1	Mean 2	Adjusted P value	Mean 1	Mean 2	Adjusted P value
SPRAY VS. VIAL	1.5	2	0.65	1	4	< 0.01^*∗*^	1	1	> 0.99
SPRAY VS. SYRINGE	1.5	3	< 0.01^*∗*^	1	3.75	< 0.01^*∗*^	1	1	> 0.99
SPRAY VS. MAD	1.5	2.25	0.21	1	4	< 0.01^*∗*^	1	4	< 0.01^*∗*^
SPRAY VS. SPRAY-SOL	1.5	3.75	< 0.01^*∗*^	1	5	< 0.01^*∗*^	1	5	< 0.01^*∗*^
SPRAY VS. RINOWASH	1.5	3.5	< 0.01^*∗*^	1	3.25	< 0.01^*∗*^	1	1.25	0.97
VIAL VS. SYRINGE	2	3	0.03	4	3.75	0.97	1	1	> 0.99
VIAL VS. MAD	2	2.25	0.97	4	4	> 0.99	1	4	< 0.01^*∗*^
VIAL VS. SPRAY-SOL	2	3.75	< 0.01^*∗*^	4	5	0.03	1	5	< 0.01^*∗*^
VIAL VS. RINOWASH	2	3.5	<0.01^*∗*^	4	3.25	0.21	1	1.25	0.97
SYRINGE VS. MAD	3	2.25	0.21	3.75	4	0.97	1	4	< 0.01^*∗*^
SYRINGE VS. SPRAY-SOL	3	3.75	0.21	3.75	5	<0.01^*∗*^	1	5	< 0.01^*∗*^
SYRINGE VS. RINOWASH	3	3.5	0.65	3.75	3.25	0.65	1	1.25	0.97
MAD VS. SPRAY-SOL	2.25	3.75	<0.01^*∗*^	4	5	0.03	4	5	0.03
MAD VS. RINOWASH	2.25	3.5	<0.01^*∗*^	4	3.25	0.21	4	1.25	< 0.01^*∗*^
SPRAY SOL VS. RINOWASH	3.75	3.5	0.97	5	3.25	< 0.01^*∗*^	5	1.25	< 0.01^*∗*^

**(b):** 

	NASOPHARYNX			NASAL CAVITY FLOOR			SUPERIOR OLFACTORY CLEFT		
	Mean 1	Mean 2	Adjusted P value	Mean 1	Mean 2	Adjusted P value	Mean 1	Mean 2	Adjusted P value
SPRAY VS. VIAL	1	2	0.03	1	4	< 0.01^*∗*^	1	1	> 0.99
SPRAY VS. SYRINGE	1	1	> 0.99	1	5	< 0.01^*∗*^	1	2.75	< 0.01^*∗*^
SPRAY VS. MAD	1	1	> 0.99	1	5	< 0.01^*∗*^	1	3.75	< 0.01^*∗*^
SPRAY VS. SPRAY-SOL	1	4.25	< 0.01^*∗*^	1	5	< 0.01^*∗*^	1	3.75	< 0.01^*∗*^
SPRAY VS. RINOWASH	1	1	> 0.99	1	4.75	< 0.01^*∗*^	1	2	0.03
VIAL VS. SYRINGE	2	1	0.03	4	5	0.03	1	2.75	< 0.01^*∗*^
VIAL VS. MAD	2	1	0.03	4	5	0.03	1	3.75	< 0.01^*∗*^
VIAL VS. SPRAY-SOL	2	4.25	< 0.01^*∗*^	4	5	0.03	1	3.75	< 0.01^*∗*^
VIAL VS. RINOWASH	2	1	0.03	4	4.75	0.21	1	2	0.03
SYRINGE VS. MAD	1	1	> 0.99	5	5	> 0.99	2.75	3.75	0.03
SYRINGE VS. SPRAY-SOL	1	4.25	< 0.01^*∗*^	5	5	> 0.99	2.75	3.75	0.03
SYRINGE VS. RINOWASH	1	1	> 0.99	5	4.75	0.97	2.75	2	0.21
MAD VS. SPRAY-SOL	1	4.25	< 0.01^*∗*^	5	5	> 0.99	3.75	3.75	> 0.99
MAD VS. RINOWASH	1	1	> 0.99	5	4.75	0.97	3.75	2	< 0.01^*∗*^
SPRAY-SOL VS. RINOWASH	4.25	1	< 0.01^*∗*^	5	4.75	0.97	3.75	2	< 0.01^*∗*^

## Data Availability

The data used to support the findings of this study are available from the corresponding author upon request.
